# Measuring Carbon Market Transaction Efficiency in the Power Industry: An Entropy-Weighted TOPSIS Approach

**DOI:** 10.3390/e22090973

**Published:** 2020-08-31

**Authors:** Jin Zhu, Huaping Sun, Nanying Liu, Dequn Zhou, Farhad Taghizadeh-Hesary

**Affiliations:** 1 College of Economics and Management, Nanjing University of Aeronautics and Astronautics, Nanjing 211106, China; zhujin1981@nuaa.edu.cn (J.Z.); dqzhou@nuaa.edu.cn (D.Z.); 2 Division of Low-carbon Economy and Environmental Regulation, Institute of Industrial Economics, Jiangsu University, Zhenjiang 212013, China; nine1084@163.com; 3 Social Science Research Institute, Tokai University, Hiratsuka-shi, Kanagawa-ken 259-1292, Japan

**Keywords:** power industry, carbon market, transaction efficiency, TOPSIS, entropy-weighted approach

## Abstract

Carbon emission control is an urgent environmental issue that governments are paying increasing attention to. Improving carbon market transaction efficiency in the context of China’s power industry is important for green growth, low carbon transmission, and the realization of sustainable development goals. We used the entropy-weighted Technique for Order Preference by Similarity to an Ideal Solution (TOPSIS) method in this empirical study to analyze the carbon market transaction efficiency of China’s power industry. The results showed that the Beijing carbon market has the highest transaction efficiency, followed by those of Guangdong Province and Shenzhen City. Hubei Province also has a relatively high carbon market transaction volume and turnover; its transaction efficiency ranks fourth. Shanghai, Tianjin, and Chongqing are the lowest-ranked regions, having carbon markets with relatively low trading volume and turnover. We, therefore, recommend that to develop a unified national carbon market, governmental agencies at all levels should equitably allocate carbon; strict regulations and penalties are also needed.

## 1. Introduction

Carbon dioxide is an important greenhouse gas implicated in global warming and sea-level rise [1]. Since it takes a long time to generate electricity from renewable energy, a solution to the problem of the massive carbon emissions generated by conventional coal and gas-fired power plants is urgently required [2,3,4]. The power industry is an indispensable resource-based industry in China, and also plays a central role in the carbon trading market. The total amount of carbon emissions and the dynamic trading of carbon emission rights are important issues for the power industry. The main purpose of carbon trading is to reduce the emission of pollutants, benefit human and environmental systems, and change the power industry from one characterized by high resource consumption to a clean industry [5,6]. Pilot regional carbon trading schemes have been conducted in China for many years, and valuable experience has been gained. However, the success of the national carbon market and quota allocation depends on the further development of the industry. Quota allocation methods have advantages and disadvantages, and no method is ideal in all situations. Each region should use a quota allocation method that is suitable based on its developmental stage and resources, with adjustments and innovations implemented as necessary.

Ref. [7] applied data envelopment analysis (DEA) to the manufacturing industries of 14 OECD countries and showed that carbon dioxide emissions are undesirable with respect to environmental performance. Lower and upper acceptable limits were also provided. Zero-sum gains data envelopment analysis (ZSG-DEA) models are designed to evaluate efficiency. When applied for resource allocation, repeated model iterations are needed [8,9]. Moreover, [10] used a non-radial ZSG-DEA model to efficiently allocate carbon dioxide emission quotas to different provinces in China, as a Pareto optimal solution. Using a proxy model, [11] showed that emission trading can provide the Chinese government and relevant decision-makers with quantitative tools to design carbon markets. Ref. [2] analyzed carbon emission reduction measures used by the power industry. Ref. [12] proposed two equilibrium models with parallel queues that could also aid decision-makers.

These studies have led to equitable carbon emission allowances, thus resolving conflicts between central authorities and power plants. A bi-level programming method is used for accurate equilibrium modeling [13]. Furthermore, [14] stated that a carbon tax serves as a proxy of the price of carbon. However, at present, determining the ideal tax rate to reduce carbon emissions is difficult. Among the various policy plans, ET is the most promising, because it is politically feasible, cost-effective, and provides economic incentives facilitating policy objectives [15,16,17,18,19]. Ref. [20] analyzed the allocation of carbon permits in a putative carbon emission trading scheme for the Australian electricity market. Ref. [21] analyzed the impact of low-emission tramcars in Japan on carbon emissions. Ref. [22] reported on energy sector planning under carbon constraints. Ref. [23] discussed the carbon trading market from the perspective of sector development, while [24] discussed incentives for carbon emission reduction in developing countries, especially carbon emission permits. Ref. [25] analyzed the feasibility and fairness of carbon emission quotas. Ref. [19] performed an efficiency analysis of carbon emission permits. Although the principles of fairness and efficiency differ, some studies assert that the principle of efficiency can, to a certain extent, be regarded as a subprinciple of fairness [26,27].

Ref. [28,29,30] discussed the implications of carbon emission trading for regional power system planning and pointed out that carbon price plays an important role in carbon emission reduction. Ref. [31] proposed a mathematical market model to analyze the short- and long-term economic benefits of green certificate trading. Furthermore, [32] stated that the price of emission permits is affected by the issuance thereof. If the allocation is strict, supply will decrease and demand will increase, which will lead to higher market prices. Moreover, [33] found that both the actual and expected energy price affect the emission price. Ref. [34] discussed the basic ideas and methods of Monte Carlo simulations for power grid planning considering uncertainties in the electricity market. Ref. [35] used a set pair theory to analyze uncertainties associated with power grid planning and established a flexible model for meeting multiple power grid planning goals. Ref. [36] discussed the implementation and operation of a carbon quota for China. Ref. [37] evoked the principle of fairness in a model based on benchmarks, the “grandfather system” and the Shapley value, to allocate carbon emission allowances among the three main power plants in Shanghai.

DEA is a representative method for efficiency evaluation that was proposed by [38]. This well-known model is widely used to optimize configurations [39,40,41,42]. For example, Ref. [43] used a ZSG-DEA model to explore the allocation and redistribution of emission allowances among 24 EU countries. Although DEA has been widely used in efficiency evaluations, and proven effective for measuring the efficiency of carbon allowance allocation, other efficiency evaluation methods are also available. For example, [44] analyzed 10 strategies for introducing emission quotas and showed that an equivalent reduction in greenhouse gases over time minimizes the economic cost. The quota system for the power industry should be improved and updated to meet current demands. At a fundamental level, carbon trading is intended to achieve efficient resource allocation [45,46,47]. Although no perfect method for quota allocation exists, the objectives of individual regions are similar. Various transaction regulations should be used to reduce emission reduction costs and optimize resource allocation. The quota model itself, policy measures, the acceptability of the quota allocation method, and ease of operation are all important factors. When fairness and efficiency are relatively well balanced, the quota price can play a role in stabilizing the carbon market.

Since 2013, a carbon trading volume of 200 million tons (4 billion Yuan) has been recorded for the seven regions in China, where pilot carbon trading schemes have been implemented (Beijing, Shanghai, Tianjin, Chongqing, Hubei, Guangdong, and Shenzhen). The quota system can affect the efficiency of the carbon market. It is, therefore, necessary to empirically test this proposition, to build an open, transparent, and smoothly operating carbon market. In the following, an empirical analysis of the transaction efficiency of the seven pilot carbon trading schemes is described, based on actual data.

## 2. Methods and Materials

### 2.1. Research Methods

The Technique for Order Preference by Similarity to an Ideal Solution (TOPSIS) is a method for solving decision-making problems commonly used in comprehensive evaluations. The basic principle of the TOPSIS method is to determine the positive ideal solution and negative ideal solution, and as well normalize the data of different indexes. The Euclidean distances between the evaluation object and the optimal and worst solutions are calculated to evaluate the advantages and disadvantages of a particular scheme. If the evaluation object is closer to the positive than the negative ideal solution, it can be considered optimal. TOPSIS can effectively eliminate the influence of differences among indexes on the results, to reflect the actual situation more directly and reliably [48].

When using TOPSIS to evaluate carbon market trading efficiency in the context of the power industry in China, determining the relative weight of different indicators is important. Two main methods are used to this end: (1) subjective weighting, which is a relatively subjective approach based on questionnaire surveys and consultations with industry experts, and (2) objective weighting, namely the entropy-weighted method, which depends on the dispersion of the data and uses the entropy value of different indexes to determine the weights; the greater the degree of dispersion of an index, the greater its impact on the evaluation [49,50,51]. Compared with subjective weighting, the entropy-weighted method can obtain more objective evaluation results. The entropy-weighted method is therefore adopted in this paper to determine the weights of different factors in the carbon trading market.

### 2.2. Evaluation Indexes

There are significant differences in the level of economic development, industrial structure, and energy structure among the seven pilot carbon trading markets in China. Therefore, operating efficiency and policies also differ among the markets. To more comprehensively analyze carbon market transaction efficiency in the seven pilot markets, quota allocation efficiency and market transaction efficiency are analyzed in this paper. Quota allocation efficiency is evaluated according to the effectiveness and stability of carbon price, while the efficiency of the trading market is evaluated based on market liquidity and regulatory mechanisms [52,53,54,55,56,57,58]. The carbon market trading efficiency evaluation index for the seven pilot markets is shown in Table 1.

#### 2.2.1. Carbon Price Effectiveness

Carbon price is associated with the short-term cost of marginal emission reduction to society as a whole, and national emission reduction targets. The validity of the carbon price is in accordance with the validity of the carbon market transaction price. A mechanism to establish the carbon price is of key importance for carbon markets. An effective and reasonable carbon price not only ensures the steady development of the economy but also encourages enterprises to innovate and implement energy-saving and emission reduction measures. In this paper, the effectiveness of the carbon price is measured based on the number of enterprises. The most efficient way of measuring carbon price in this study is to use (A1).

A1 as defined already is the number of emission-controlled enterprises within the carbon market of each pilot market. Generally, when there are more enterprises in the carbon emission trading system, the carbon market is more effective, such that the potential for carbon emission reduction becomes greater.

#### 2.2.2. Carbon Price Stability Index

Carbon price stability refers to the degree of fluctuation in the carbon market price. A stable carbon price is not only conducive to the formulation of policies and regulations pertaining to carbon emission reduction but is also important for strategic decision-making by enterprises within the carbon trading system. This can improve the stability of the carbon trading market and the efficiency of quota allocation. Carbon price stability in this paper is determined based on a “carbon price index” (A2), using the method of [59].

We first need to determine the stability of the weighted carbon price $\bar{p}$. This is done by obtaining the trading price of carbon within a certain trading period to derive the weighted carbon price for each pilot market, using the following formula:

(1) $$\bar{p}=\frac{\sum p_{i}w_{i}}{\sum w_{i}}$$

where *p_i_* is the transaction price on the *i*-th trading day and *w_i_* is the trading volume on the *i*-th trading day. Within a certain trading period, it is also necessary to set a reasonable floating range r (10%) for the carbon trading price, that is, the total number of transactions within the carbon trading price range of $\bar{p}$ (1 ± 10%), divided by the total trading volume. The ratio between the two values is the “carbon price stability index”, *β_i_*. The larger the value of *β_i_*, the more representative the weighted carbon price is within this trading period, and the more stable the carbon price is. Ultimately, the carbon price index $\eta_{i}$ is the product of the weighted carbon price and the carbon price stability index *β_i_* within a certain trading period, calculated as follows:

(2) $$\eta_{i}=\bar{p}\cdot \beta_{i}$$

#### 2.2.3. Market Liquidity Index

The market liquidity index represents the efficiency of the carbon trading market. The key to determining the liquidity of the carbon market is to first measure its size. The most intuitive measurement of the size of the carbon market is the carbon market turnover (B2): the larger the total transaction volume and total transaction amount, the greater the liquidity of capital, and thus the greater the liquidity of the carbon market. If the liquidity of the carbon market is relatively low, it is difficult to improve its efficiency.

The trading activity of the carbon market (B3) is another important indicator of the liquidity of the carbon market. The total volume of carbon trading in the trading market of each pilot market was divided by the total quota of the carbon market in each pilot area.

#### 2.2.4. Regulatory Mechanism

Although carbon quota allocation and carbon trading mechanisms are the main factors that restrict or expand the carbon market, the inclusion of enterprises is the main driver of trading efficiency. An effective regulatory mechanism is also indispensable for carbon trading systems. This study used the number of third-party verification institutions (B4) as a measure of the effectiveness of the regulatory mechanism of the pilot carbon trading markets.

A third-party organization is responsible for monitoring greenhouse gas emissions and verifying reports submitted by “emission-controlled enterprises” operating within the carbon emission trading system. Emission-controlled enterprises and third-party verification institutions are responsible for the authenticity, accuracy, and integrity of the reported data, which is important for maintaining fairness and transparency within a carbon market and guaranteeing its efficiency.

### 2.3. Model Setup

#### 2.3.1. Normalization of the Original Data

If there are m evaluation objects and n evaluation indexes in the efficiency evaluation system, an efficiency evaluation matrix, *X* = (*x_ij_*) *m* × *n*, *i* = 1, 2, *m*; *j* = 1, 2 …, *n*, can be established.

In using the TOPSIS method for efficiency evaluation, the direction of change must be consistent among all indicators. However, since the efficiency evaluation indexes in this paper are all positive indicators, this step is omitted. Hence the data is normalized, as follows:

(3) $$Z_{ij}=\frac{X_{ij}}{\sqrt{\sum_{i=1}^{m}X_{ij}^{2}}}$$

#### 2.3.2. Entropy-Weighted Method

The Entropy-weighted method is used to determine the weight of each index. First, the entropy value of the *j*th index is calculated, as follows:

(4) $$e_{j}=-k\overset{m}{\underset{i=1}{\sum }}Z_{ij}\ln(Z_{ij})$$

where, $k=\frac{1}{\ln(m)}$

Then, the utility information entropy is calculated, as follows:

(5) $$c_{j}=1-e_{j},\;j=1,\;2,\;\ldots ,\;n$$

The entropy weight is then calculated:

(6) $$w_{j}=\frac{c_{j}}{\sum_{j=1}^{n}c_{j}},\;j=1,\;2,\;\ldots ,\;n$$

Then, the construction of a standardized weight matrix is computed as follows:

(7) $$r_{ij}=w_{j}\cdot Z_{ij}$$

#### 2.3.3. Determination of Positive and Negative Ideal Solutions

We have:

$$r_{j}^{+}=\underset{1\leq i\leq m}{max}\left\{r_{ij}\right\}\;r_{j}^{-}=\underset{1\leq i\leq m}{min}\left\{r_{ij}\right\},\;j=1,\;2,\;\ldots ,\;n$$

Thus, the positive ideal solution $r^{+}=\left(r_{1}^{+},r_{2}^{+},\ldots ,r_{n}^{+}\right)$ is the maximum value of the *j*th index of the evaluation object, and the negative ideal solution $r^{-}=\left(r_{1}^{-},r_{2}^{-},\ldots ,r_{n}^{-}\right)$ is the minimum value of the *j*th index of the evaluation object.

#### 2.3.4. Calculating the Euclidean Distance

The Euclidean distance is the distance between each evaluation object and the optimal solution *r*^+^ and worst solution *r*^−^. The formula is:

(8) $$d_{i}^{+}=\sqrt{\overset{n}{\underset{j=1}{\sum }}{\left(r_{ij}-r_{j}^{+}\right)}^{2}}\;d_{i}^{-}=\sqrt{\overset{n}{\underset{j=1}{\sum }}{\left(r_{ij}-r_{j}^{-}\right)}^{2}}$$

#### 2.3.5. Degree of Closeness between Object and Positive Ideal Solution

The degree of closeness between each object and positive ideal solution is computed by using the formula:

(9) $$f_{i}=\frac{d_{i}^{-}}{d_{i}^{-}+d_{i}^{+}}$$

The greater the degree of closeness *f_i_*, the farther the evaluation objects are from the negative ideal solution, and the closer they are to the positive ideal solution.

#### 2.3.6. Sorting by Closeness

According to *f_i_*, the greater the degree of closeness, the higher the carbon trading efficiency of the pilot market, and vice versa.

## 3. Results and Discussion

Based on the data of the seven pilot carbon trading markets in China from 2014 to 2018, a carbon trading efficiency evaluation system was constructed based on six factors: (1) the number of emission-controlled enterprises (A1), (2) the carbon price index (A2), (3) the total number of carbon market transactions (B1), (4) the total carbon market turnover (B2), (5) the trading activity (B3), and (6) the number of third-party verification institutions (B4). Since the data are for the carbon trading pilot markets as a whole and do not involve the power industry in each region, we took the ratio of the annual industrial output value of the electric power, heat production, and supply industry, and the total industrial output value of each pilot area as the proportion of the carbon trading data of the power industry in the overall carbon trading data for each pilot area. The relevant data of each index comes from the statistical yearbook of 7 carbon pilot cities and provinces, and the websites of various carbon emission exchanges.

According to the formula for the entropy-weighted method, the weights of the carbon trading efficiency evaluation indexes for each pilot market were calculated. The weights of the various indexes are shown in Table 2.

The weights for quota allocation efficiency and carbon market transaction efficiency were not significantly different, at 0.42 and 0.58, respectively.

The weight for carbon price effectiveness was 0.2371, which is higher than the value for the carbon price stability index. The weight for market liquidity was 0.3969 and that for the regulatory mechanism was 0.18.

The number of emission-controlled enterprises (A1), carbon price (A2), and number of third-party verification institutions (B4) have the largest weights, of 0.2371, 0.1861 and 0.1800, respectively. The data indicate that increasing the number of emission-controlled enterprises, strengthening carbon trading regulatory mechanisms, and maintaining carbon price stability can improve the efficiency of the carbon trading market.

After the weight of each index has been determined, the positive ideal solution and negative ideal solution of each index can be obtained by constructing a standardized weight matrix. The distances between each index and the positive and negative ideal solutions are then calculated, based on which the carbon trading efficiency of the seven pilot markets can be determined. The results are shown in Table 3:

Using the entropy-weighted TOPSIS method, the carbon trading efficiencies of the seven pilot markets were obtained. Overall, Beijing had the highest carbon trading efficiency, ranking first between 2014 and 2017 and second in 2018. Beijing also had the most emission-controlled enterprises (954 enterprises in 2015). In the same year, Shenzhen had 636 enterprises and Chongqing had 230. The number of emission-controlled enterprises in the other areas was only more than 100. Enterprises in Beijing with annual carbon dioxide emissions of 5000 tons or more (including direct and indirect emissions) are subject to emission control. Except for Shenzhen, which has annual carbon emissions of 3000 tons or more, all areas had annual carbon emissions of 10,000 tons or more. The high efficiency of the carbon trading market in Beijing is associated with a wide range of enterprises and strictness of the controls.

From 2014 to 2018, the carbon trading efficiency ranking of the Guangdong power industry showed a steady upward trend, ranking third in 2014, second between 2015 and 2017, and first in 2018. The carbon trading efficiency of the Shenzhen power industry was largely stable throughout the study period. In 2018, the total carbon trading volume of Guangzhou reached 28.362 million tons, thus ranking first, and representing a 21.33-fold increase compared with 2014 (1,270,300 tons). In 2018, the total carbon trading volume of Guangzhou was 12.6795 million tons, thus ranking second and being 6.9 times as large as that in 2014. The trading activity of the Shenzhen carbon market was ranked first throughout the study period. Trading activity improves carbon market liquidity, vitality, and efficiency. From 2014 to 2018, the number of third-party verification institutions in Guangdong Province was high; in 2018, the number was 35, while that of Shenzhen City was also high at 26.

From 2014 to 2017, the carbon trading efficiency of the Hubei power industry was ranked fourth but dropped to sixth in 2018. Hubei Province also had a high trading volume and turnover (12.3344 million tons in 2018). The peak carbon trading volume for Hubei province occurred in 2015, at 13.9041 million tons (total transaction amount = 347.409 million Yuan; 148.78 times higher than that of Chongqing). The carbon trading efficiency of Hubei Province fell to sixth place in 2018, largely due to the instability of its carbon price. In 2018, the weighted carbon price of Hubei Province was 20.55 Yuan per ton. Only 7.44% of the trading volume was in the floating range of 18.49–22.60 Yuan per ton, while in 2017, 61.38% of the trading volume was in the floating range of the weighted carbon price. Instability in the carbon price impacts on trading efficiency.

The carbon trading efficiency of the power industries in Shanghai and Tianjin was low but showed a rising trend over the study period. In Shanghai, there is a unique 3-year quota and cross-year carryover thereof; any outstanding quota of the current year can be used for trading in the following 2 years, which leads to the continuous occurrence in July and August after the performance period, continued no trading volume. The number of emission-controlled enterprises in Tianjin was the smallest among the seven pilot markets. This is because the market entry threshold for emission-controlled enterprises is high, at annual carbon emissions of more than 20,000 tons. Tianjin also has the least severe punishments and penalties for violating carbon trading rules. Emission-controlled enterprises that do not adhere do not pay a fine, nor is any violation logged in the credit reporting system. However, the enterprise is required to address any breach within a specified period and becomes ineligible for supportive governmental policies after 3 years. In other areas, breach of contract leads to credit investigations, deduction of the outstanding part of the year’s quota from that of the following year, and a fine of tens of thousands of Yuan. The lack of any strong punishment and penalties increases the likelihood of contract breaches by emission-controlled enterprises, which affects the efficiency of the carbon trading market.

The carbon trading efficiency of the Chongqing power industry was stable over the study period, i.e., it was ranked seventh (last) throughout (except for 2014; ranked sixth). Except for 2017, Chongqing had a trading volume of fewer than 1 million tons throughout the study period. In 2018, Chongqing’s carbon trading volume was only 269,400 tons (1,174,800 Yuan). Moreover, the carbon trading volume in Chongqing is mostly concentrated before and after the performance period, and there are several consecutive zero trading volumes in the nonperformance period. In 2014, only one carbon transaction was completed, and in 2015 and 2016, there were only 27 and 38 trading days, respectively. The fluctuation of the carbon price in Chongqing over the study period was large. Between 2014 and 2018, the highest average carbon price in Chongqing was 47.52 Yuan per ton and the lowest was 1 Yuan per ton. The instability of the carbon price greatly weakened the market regulatory function of the carbon price, resulting in low carbon market transaction efficiency. The Chongqing carbon trading market was implemented within a relatively short period, and its carbon trading mechanism is imperfect. Emission-controlled enterprises in the region are concerned with performance and lack awareness of the importance of emission reduction. Carbon market participation and turnover are low, which undermine carbon trading efficiency.

## 4. Conclusions and Policy Recommendations

### 4.1. Conclusions

The trading volume and performance of the pilot carbon markets in China have been steadily increasing, such that the total amount and intensity of carbon emissions have decreased. The positive effects of the carbon market with respect to the control of greenhouse gas emissions are clear, and valuable experience has been gained toward a unified, national carbon emission trading market in China. According to the evaluation results, the trading efficiency is highest for the carbon market in Beijing, followed by Guangdong Province, Shenzhen City, Hubei Province, Shanghai, Tianjin, and, in last place, Chongqing. The trading volume, turnover, and efficiency of the carbon market in the latter three regions are low.

### 4.2. Policy Recommendations

Minimizing the cost of emission reduction can serve as an incentive for emission-controlled enterprises to participate in carbon trading. The production and operations of emission-controlled enterprises are restricted by regional emission reduction policies and fines. Through transactions on the carbon market, such enterprises can minimize their emission reduction costs. For institutions and individuals, the main purpose of participating in the market is of course to obtain investment income; however, their participation renders the carbon market more active. Therefore, a rational incentive for participating in the carbon market is important. Based on the above discussion, we make the following policy recommendations.

First, before implementing carbon trading schemes, governmental agencies at all levels should distribute carbon quotas equitably. The carbon trading system for the power industry has changed the trading mechanism of power enterprises. The constraints and incentives associated with carbon quotas are derived from regulatory agencies and institutions. As well as achieving emission reduction goals, enterprises are also encouraged to improve their technology and energy resource utilization, given the competitive nature of the carbon trading market. Second, strict supervision and punishments are necessary for the effective operation of the carbon market. Penalties discourage carbon market participants from producing excessive emissions and act synergistically with incentives. In turn, this promotes fair and rational quota allocation, maintains order with respect to market transactions, and encourages improved behavior by high-emission enterprises. The carbon trading market also relies on verification institutions, consulting institutions, members of the general public, etc., to reduce market trading risk. High-quality professional services greatly improve the trading efficiency of regional power carbon trading markets in China.

Due to the limitation of data availability, this study only studies the evaluation of carbon trading efficiency in seven pilot markets in China. In the future, with the establishment of China’s unified national market, we will conduct more detailed data empirical tests.

## Figures and Tables

**Table 1:** Carbon market transaction efficiency evaluation indexes.

Primary Outcome Measures	Secondary Outcome Measures	Factors	Unit
Quota allocation efficiency	Carbon price effectiveness	Number of emission-controlled enterprises (A1)	%
	Carbon price stability	Carbon price index (A2)	%
Market transaction efficiency	Market liquidity	Total number of carbon market transactions (B1)	Ten thousand tons
		Total carbon market turnover (B2)	Ten thousand Yuan
		Trading activity (B3)	%
	Regulatory mechanism	Number of third-party verification institutions (B4)	N

Source: Authors’ compilation.

**Table 2:** Weight of each evaluation index in the period 2014–2018.

Primary Indexes	Secondary Indexes	Index Code	2014	2015	2016	2017	2018	*w_j_*
Quota allocation efficiency	Carbon price effectiveness	A1	0.3157	0.3227	0.1911	0.1875	0.1687	0.2371
	Carbon price stability	A2	0.0593	0.2233	0.2823	0.2424	0.1230	0.1861
Carbon market transaction efficiency	Market liquidity	B1	0.0299	0.0753	0.1509	0.1848	0.2369	0.1356
		B2	0.0874	0.0838	0.1330	0.1548	0.1642	0.1246
		B3	0.2654	0.1284	0.1175	0.0510	0.1210	0.1367
	Regulatory mechanism	B4	0.2423	0.1665	0.1253	0.1796	0.1861	0.1800

Source: Authors’ calculation.

**Table 3:** Carbon transaction efficiency rankings of the seven pilot markets for the period 2014–2018.

Pilot Market	Values					Ranking				
	2014	2015	2016	2017	2018	2014	2015	2016	2017	2018
Beijing	0.9974	0.9508	0.7776	0.6760	0.5657	1	1	1	1	2
Tianjin	0.0537	0.0556	0.0199	0.0456	0.1005	7	5	6	6	5
Shanghai	0.0696	0.0309	0.0291	0.0603	0.1129	5	6	5	5	4
Hubei	0.1124	0.1539	0.0745	0.0706	0.0758	4	4	4	4	6
Chongqing	0.0572	0.0144	0.0153	0.0259	0.0024	6	7	7	7	7
Guangdong	0.2256	0.2040	0.3711	0.5045	0.6490	3	2	2	2	1
Shenzhen	0.2441	0.1750	0.1977	0.0839	0.1734	2	3	3	3	3

Source: Authors’ calculation.
